# HomeoDB2: functional expansion of a comparative homeobox gene database for evolutionary developmental biology

**DOI:** 10.1111/j.1525-142X.2011.00513.x

**Published:** 2011-11-21

**Authors:** Ying-fu Zhong, Peter W H Holland

**Affiliations:** Department of Zoology, University of OxfordSouth Parks Road, Oxford OX1 3PS, UK

## Abstract

Homeobox gene database (HomeoDB), a manually curated database of homeobox genes and their classification, has been well received since its release in 2008. Here, we report HomeoDB2, an expansion and improvement of the original database that provides greater functionality for the user. HomeoDB2 includes all homeobox loci from 10 animal genomes (human, mouse, chicken, frog, zebrafish, amphioxus, nematode, fruitfly, beetle, honeybee) plus tools for downloading sequences, comparing between species and BLAST searching. HomeoDB2 provides a resource for studying the dynamics of homeobox gene evolution, and is freely accessible at http://www.homeodb.zoo.ox.ac.uk

## INTRODUCTION

Homeobox genes are found across eukaryotes but are most diverse in animal genomes. A robust evolutionary classification of metazoan homeobox genes has been established comprising 11 gene classes and over 100 gene families (Edvardsen et al. 2005; Ryan et al. 2006; Holland et al. 2007). “Gene classes” are defined using motifs outside the homeodomain or as large clades of related genes; “gene families” comprise all genes descended from a single gene in the common ancestor of bilaterian animals, although additional gene families have been erected for genes of unknown orthology. Using this classification scheme, we developed a manually curated database of homeobox genes: HomeoDB (Zhong et al. 2008). In its first release, HomeoDB covered three species and gave basic information for each homeobox locus, plus links to other databases and descriptions of each gene class and family. The database was designed to make comparison between orthologues and paralogues easy and clear. Since its first release, HomeoDB has been used widely and accessed at a high rate; for example, a mean of 163 visits/day (455 page views/day) between 10/2010 and 07/2011, from 50 countries. Here, we draw attention to a series of updates and significant enhancements to HomeoDB.

## METHODS

In HomeoDB2, we used genome sequence data of *Homo sapiens* Build 37.2 (GRCh37.p2), *Mus musculus* Build 37.1 (C57BL/6J), *Gallus gallus* Build 2.1 (Gallus_gallus-2.1), *Danio rerio* (Zv9), *Xenopus (Silurana) tropicalis* Build 1.1 (v4.2), *Drosophila melanogaster* (Release 5.30), *Tribolium castaneum* (Tcas_3.0), and *Apis mellifera* (Amel_4.5) from NCBI FTP server (ftp://ftp.ncbi.nih.gov/genomes/); *Caenorhabditis elegans* (WS220) from WormBase (http://www.wormbase.org/); *Branchiostoma floridae* (v2.0) from JGI (http://genome.jgi-psf.org/Brafl1/Brafl1.home.html). Sequence searches and locus identification followed Zhong and Holland (2011). The HomeoReg dataset was collected from the literature; models of hybridization were edited from results of RNAhybrid (Rehmsmeier et al. 2004). HomeoDB2 was recoded through an Apache+Perl+MySQL web application technology base on Model-View-Controller design pattern.

## RESULTS AND DISCUSSION

From May 2008 to July 2011, the species coverage of HomeoDB was enlarged. By version 3.5.2, HomeoDB included human, mouse, chicken, zebrafish, frog (*X. tropicalis*), amphioxus (*B. floridae*), fruitfly (*D. melanogaster*), beetle (*T. castaneum*), honeybee (*A. mellifera*), and a nematode (*C. elegans*). As new genome assemblies were released for particular species, compilations and classifications were refined and updated. At the request of the HUGO Gene Nomenclature Committee (HGNC), each human gene page has also been bidirectionally cross-linked to its HGNC gene symbol report. In its latest release, HomeoDB contains 1929 homeobox loci, comprising 1763 probable genes, 125 probable pseudogenes, and 41 loci with undefined annotation, classified into 127 gene families in 11 classes, plus several unclassified groups.

To increase functionality, we have incorporated several new features and release these as a new version, HomeoDB2. First, we have integrated a standalone BLAST server. Users can input a nucleic acid or amino acid sequence as a query and use blastx or blastp to search the HomeoDB2 dataset. The advantages over searching complete genomic or RefSeq databases are speed and immediate access to gene family data. We recommend doing BLAST only as an initial clue to a gene's identity and following this with both molecular phylogenetics and analysis of synteny. Second, HomeoDB2 includes a “Compare” function, which allows users to select any species in the database to compare, either across all homeobox genes or subsets of the gene classification. This allows users to home in on gene duplications and gene losses for further study. Third, HomeoDB2 includes a “Download” function so that users can download homeodomain sequences from any species, classes, or families (or combinations) in FASTA format.

The core role of HomeoDB2 remains the classification of coding genes, but we have also initiated HomeoReg as a subdataset of HomeoDB2. This aims to collect information on experimentally demonstrated regulatory interactions between noncoding RNA molecules (e.g., miRNA) and homeobox genes. Only those relevant to homeobox genes in HomeoDB2 are included; these comprise 46 interactions at present. These regulators are displayed with their target homeobox genes showing hybridization information.

Ultimately, it would be ideal to include in HomeoDB2 all species for which complete genome sequence data are available. Quality of sequence and assembly is not uniform, however, which makes comprehensive identification and annotation of homeobox sequences impossible for many genomes. By focusing on animal models with well-assembled and annotated genomes, HomeoDB2 provides a resource suitable for investigating the dynamics of homeobox gene evolution along major evolutionary lineages. For example, we have exploited HomeoDB for a comprehensive comparison between human and mouse genomes, revealing far more homeobox gene loss in the rodent evolutionary lineage than in the primate lineage (Zhong and Holland 2011). HomeoDB2 is freely available at http://homeodb.zoo.ox.ac.uk.
